# Orbital Apex Syndrome: A Case Series in a Tertiary Medical Center in Southern Taiwan

**DOI:** 10.3389/fmed.2022.845411

**Published:** 2022-03-08

**Authors:** Peng-Hsuan Lee, Shih-Chieh Shao, Wan-Ju Annabelle Lee

**Affiliations:** ^1^Department of Ophthalmology, National Cheng Kung University Hospital, College of Medicine, National Cheng Kung University, Tainan, Taiwan; ^2^Department of Pharmacy, Keelung Chang Gung Memorial Hospital, Keelung, Taiwan; ^3^School of Pharmacy, Institute of Clinical Pharmacy and Pharmaceutical Sciences, College of Medicine, National Cheng Kung University, Tainan, Taiwan; ^4^Department of Ophthalmology, Chi Mei Medical Center, Tainan, Taiwan; ^5^Department of Optometry, Chung Hwa University of Medical Technology, Tainan, Taiwan

**Keywords:** orbital apex syndrome, OAS, ophthalmoplegia, proptosis, case series

## Abstract

**Background:**

Orbital apex syndrome (OAS) is a rare ocular complication following by infection, inflammation, trauma, neoplasms, and vascularity. The epidemiological features of OAS remained limited, so this study aimed to present ophthalmic clinical features, determine the causes to evaluate the visual prognosis of orbital apex syndrome (OAS) patients in Taiwan.

**Methods:**

This was a retrospective study by reviewing the electronic medical records from National Cheng Kung University Hospital in Taiwan during 2017–2019. We included patients diagnosed with OAS to review their ocular symptoms and signs, visual acuity, ocular images, etiologies, treatment and visual prognosis.

**Results:**

Twenty cases (mean age: 65.55 ± 13.06; male: 75%) with the diagnosis of OAS were included in this study. All patients presented as unilateral involvement, but the initial ocular presentations and etiologies varied. For example, blurred vision was reported in 80% of these patients, and tumor-related compression (55%) and infection (15%) were the most frequent causes for the OAS. After the follow-up, we found 35% of patients' visions declined or worsened to the blindness, 15% of patients' visions remained stable, 20% of patients' visions had mild improvement, and 35% of patients' visions were not measured because of debilitating clinical condition. We identified three OAS patients with mortality (15%), and all of them were attributed to the underlying malignancies.

**Conclusion:**

The clinical magnifications and etiologies of OAS are heterogeneous in Taiwan. Our findings indicated the tumor-related compression is the most frequent causes of OAS in Taiwan, and it is also related to poor clinical outcomes.

## Introduction

The orbital apex is an area that includes structures from the tendinous annulus of Zinn, optic nerve, and ophthalmic artery through the optic canal (1). Orbital apex syndrome (OAS) is defined as a collection of dysfunctions involving multiple cranial nerves (CNs), including the optic, oculomotor, trochlear, and abducens nerves, and the ophthalmic branch of the trigeminal nerve. OAS results from disease processes occurring in the region of the optic canal, superior orbital fissure, or cavernous sinus. The hallmarks of OAS are vision loss from optic neuropathy and ophthalmoplegia involving multiple CNs (2).

OAS can be attributed to a variety of clinical conditions, such as neoplastic diseases (3, 4) (e.g., local tumor invasion and distant metastatic tumors), inflammatory processes (5) (e.g., systemic lupus erythematosus, sarcoidosis, and Tolosa-Hunt syndrome), infections (6–8) (e.g., bacterial or fungal sinusitis and herpes zoster), traumatic injuries (9) (e.g., iatrogenic and craniomaxillofacial fracture), and vascular conditions (10) (e.g., carotid cavernous fistula, carotid cavernous aneurysms, and cavernous sinus thrombosis).

Detailed patient history with a review of the systems and thorough physical examination assessing neurological dysfunctions is important in narrowing the differential diagnosis. Imaging modalities, such as computed tomography (CT), magnetic resonance imaging (MRI), and endoscopy, are usually warranted. Surgical biopsy for histopathological examination is sometimes required for definitive diagnosis. Treatment should be directed toward the underlying process and usually involves a multidisciplinary approach.

The severity and prognosis of OAS vary widely due to the large variety of disease conditions. Furthermore, disease prevalence varies across countries and continents. Given the lack of data regarding the ophthalmic presentations of patients with OAS in the Chinese population, we aimed to delineate the clinical features, possible etiology, potential management, visual prognosis, and systemic prognosis of OAS in Taiwan.

## Method

### Patient Enrolment

Two ophthalmologists (PH Lee and WA Lee) retrospectively identified patients diagnosed with OAS by reviewing the electronic medical records from the National Cheng Kung University Hospital, a tertiary referral hospital in Taiwan, between January 1, 2017, and December 31, 2019.

### Medical History

We performed descriptive analyses by recording the patients' demographics (e.g., sex and age), disease characteristics (e.g., laterality of the involved eyes, clinical manifestations, imaging findings, histopathologic results, initial and final best-corrected visual acuity, and etiologies), treatment patterns (e.g., surgery, medications, and radiotherapy), prognosis, and mortality.

### Diagnosis

Diagnoses of OAS were based on the patients' clinical signs and symptoms. Medical imaging, such as CT and MRI, was performed in all patients except for in case No. 18 due to loss to follow-up. Furthermore, histopathologic specimens were obtained from certain patients who underwent surgical intervention.

### Prognosis

Visual outcomes and mortality were evaluated in this study. The best-corrected visual acuity was assessed using the Landolt C chart with autorefraction. A visual acuity worse than 20/200 was deemed poor in this study.

### Statistical Analysis

Categorical variables are presented as number and percentage values and were analyzed using Fisher's exact test due to the relatively small sample size. Continuous variables are presented as the mean and standard deviation (SD) and were analyzed using Student's *t-test*. A *P-*value of <0.05 was considered the threshold for statistical significance. The study data were analyzed using SPSS V.20.0 (IBM; Chicago, Illinois, USA) and the Excel software program (Office 2010, Microsoft Corp, Redmond, WA, USA). This study was conducted in accordance with the Declaration of Helsinki and was approved by the Institutional Review Board of the National Cheng Kung University Hospital, Taiwan (IRB A-ER-109-077).

## Results

### Patient Characteristics

We included twenty patients with OAS in this study. Their mean age was 65.6 (SD: 13.1) years, and most of them were male (75%). The follow-up period of these patients ranged from 0 to 24 months, with a mean of 8.8 (SD: 8.6) months.

### Disease Characteristics

On initial presentation, all patients had single-eye involvement of OAS with a wide variety of ocular manifestations (**Table 2**). Initially, the most common manifestations in these patients included blurring of vision (80%), ptosis (55%), and proptosis (40%). Other less frequent symptoms included diplopia, headache, facial numbness, redness, and periorbital pain.

The limitation of extraocular movement was observed in 90% of the patients, and 80% had documented CN III, IV, or VI involvement. The ophthalmic division of CN V was affected in three (15%) patients, among whom one also had maxillary division involvement. Hertel exophthalmometer measurements were taken in 25% of the patients and showed an average of 2.8 ± 1.5 mm proptosis on the lesion side. Ocular hypertension was recorded in 20% of the cases.

The initial visual acuity of the twenty patients was evaluated using the Landolt C chart and ranged from no light perception to 20/30. Half (ten patients) of the patients had a visual acuity worse than 20/200 upon presentation, and within which half of them (five patients) had no light perception. Positive relative afferent papillary defects were detected in 60% of the patients.

### Diagnostic Tools

All the patients underwent imaging investigations, including eight patients with CT only, five patients with MRI only, and six patients with both CT and MRI. However, one patient received no imaging evaluation due to loss to follow-up.

Eleven patients underwent tissue biopsy for definitive diagnosis, and the histopathologic results showed infection in three patients and neoplasm in eight. Six patients were diagnosed by typical imaging examinations along with a comprehensive systemic work-up and detailed medical history review, which revealed that three had autoimmune-related inflammatory processes, two had vascular tumors, and one had a trauma-related condition. Direct invasion or metastatic malignant neoplasm was presumed to be the cause of OAS in three cases in which tissue biopsy was not performed in view of the surgical risks and poor general condition of the patients due to advanced cancer. Therefore, the diagnosis was made based on typical imaging, a medical history review, or previous evidence of central nervous system metastasis in other locations.

### Etiology of OAS

**Figure 1:**
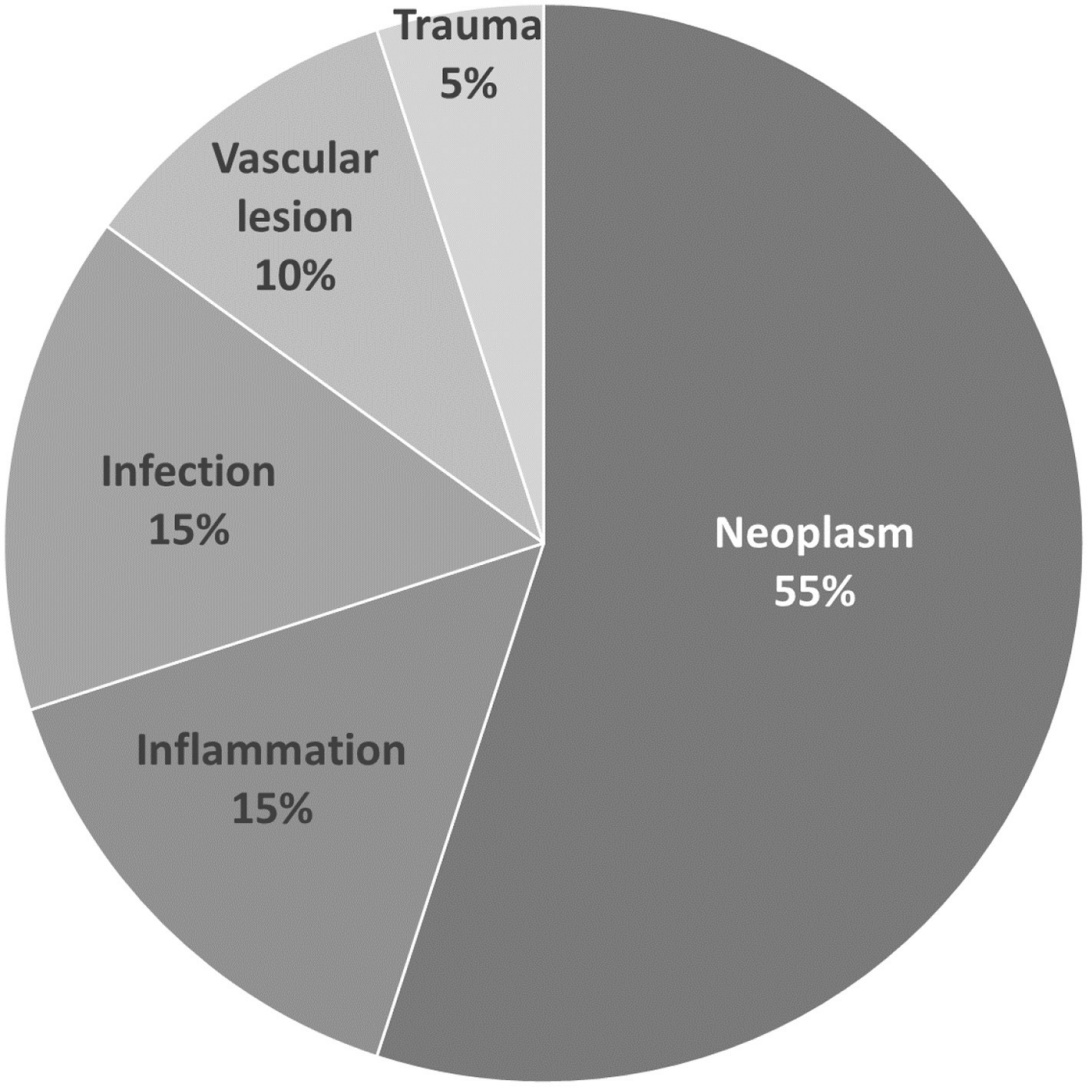
Etiology of orbital apex syndrome.

Among the twenty patients, neoplasm was the leading cause of OAS, accounting for 55% (11/20), followed by inflammatory causes accounting for 15% (3/20), infectious causes for 15% (3/20), vascular causes for 10% (2/20), and trauma-related causes for 5% (1/20) (Figure 1).

Among the eleven patients with neoplastic causes, eight were diagnosed by a histopathology examination, and three by typical imaging only. The neoplasms leading to OAS observed in our study were found to be malignant in 90.9% (10/11) of cases, with only one meningioma being considered benign. The pathophysiology of malignant neoplastic causes of OAS in our study could be categorized into direct local invasion from head and neck cancers (6/10) or metastasis from distant sites (4/10). The pathology of metastatic tumors included breast cancer (2), bladder cancer (1), and prostate cancer (1). As for advanced head and neck cancer, tongue (1), palate (1), buccal (2), nasopharyngeal (1), and paranasal sinus cancers (1) were diagnosed. The different etiologies for OAS in our study are shown in Table 1; Figure 1.

**Table 1:** Basic information of each patient with orbital apex syndrome.

**No**.	**Age**	**Sex**	**OD/OS**	**Cause**	**Clinical manifestation**										**Initial VA**	**Final VA**
					**Redness**	**Pain**	**Blurred vision**	**Headache**	**Facial numbness**	**Elevated IOP**	**Proptosis**	**Ptosis**	**Limited EOM**	**RAPD**		
1	89	M	OS	Unspecified inflammation	–	–	+	–	+	–	–	+	+	–	20/50	20/63
2	80	F	OD	Systemic lupus erythematosus	+	+	–	–	–	+	–	–	+	–	20/63	20/63
3	75	M	OD	Urothelial carcinoma with bone metastasis	–	–	+	–	–	–	+, 3 mm	+	+	+	NLP	LP
4	47	M	OD	Tongue cancer with local invasion	–	–	+	–	–	+	+		+	+	20/50	20/32
5	83	M	OS	Fungal sinusitis	–	+	+	+	–	–	+, 2 mm	+	+	–	20/200	LP
6	52	M	OD	Fungal sinusitis	–	+	–	–	–	–	–	–	–	–	20/63	20/50
7	69	M	OS	Prostate cancer with brain metastasis	–	–	–	+	–	–	+, 1 mm	+	+	–	20/63	N/A
8	60	M	OS	Buccal cancer with local invasion	+	–	+	+	–	+	–	–	+	+	20/100	N/A
9	54	F	OD	Breast cancer with brain metastasis	–	–	+	+	–	–	–	–	+	+	NLP	NLP
10	55	M	OD	Carotid carvenous fistula	+	–	+	–	–	–	+, 5 mm	+	+	+	20/32	20/20
11	57	M	OD	NPC with local invasion	–	–	+	–	+	–	+	+	–	–	CF	NLP
12	59	M	OD	Palatal cancer with local invasion	–	–	+	–	–	–	+		+	+	20/2000	LP
13	75	F	OS	Breast cancer with brain metastasis	–	–	+	–	–	–	–	+	+	+	CF	NLP
14	75	F	OD	Meningioma	–	–	+	–	–	–	+	–	+	–	LP	N/A
15	57	F	OD	Cavernous ICA out-pouch	–	–	+	+	+	–	–	+	+	–	20/32	20/400
16	84	M	OS	Trauma	–	–	+	–	–	–	–	–	+	+	LP	N/A
17	61	M	OD	Sinusitis	–	–	–	–	–	+	+, 3 mm	+	+	+	20/100	20/200
18	69	M	OD	suspected GPA	–	–	+	–	+	–	–	+	+	+	NLP	N/A
19	42	M	OS	Buckle cancer with local invasion	+	–	+	–	–	–	+	–	+	+	NLP	N/A
20	69	M	OS	Sinus cancer with local invasion	–	–	+	–	–	–	–	+	+	+	NLP	NLP

*IOP, intraocular pressure; EOM, extraocular motility, RAPD, relative afferent pupillary defect; LP, positive light perception; NLP, no light perception, GPA, granulomatosis with polyangiitis; OD, Oculus Dexter, right eye; OS, Oculus Sinister, left eye; CF, counting finger; HM, hand motion; NPC, nasopharyngeal carcinoma; ICA, internal carotid artery; N/A, not applicable*.

*Mean of age = 65.55*.

*The amount of proptosis was quantified by Hertel exophthalmometer*.

*Cases No. 3, No. 4 and No. 7 died within 1 year after orbital apex syndrome occurred*.

### Treatment

Treatment options were determined based on the definite etiology and patients' medical conditions. Infectious causes were treated with surgical debridement and antimicrobial therapy, inflammatory causes were treated with systemic corticosteroids with dose of intravenous 1 g of methylprednisolone for consecutive 3 days followed by oral prednisolone with dose of 1 mg/kg of body weight with slow tapering, vascular causes were treated with endovascular embolization, and neoplastic causes were treated with surgical resection, chemotherapy, or radiotherapy. In our study, thirteen patients received treatment, while seven patients only received palliative management. Of those who received no specific treatment for OAS, only one patient experienced improvement in visual acuity. Of those who received various treatments aimed at the underlying etiologies, six experienced preserved or improved visual acuity.

Among patients with neoplastic causes, three patients underwent surgical resection or debulking of the tumor masses, two received radiotherapy with total doses around 20–24 Gray in multiple fractions, two received combined radiotherapy and chemotherapy, one received chemotherapy alone, and three received no further treatment.

### Visual Prognosis

**Figure 2:**
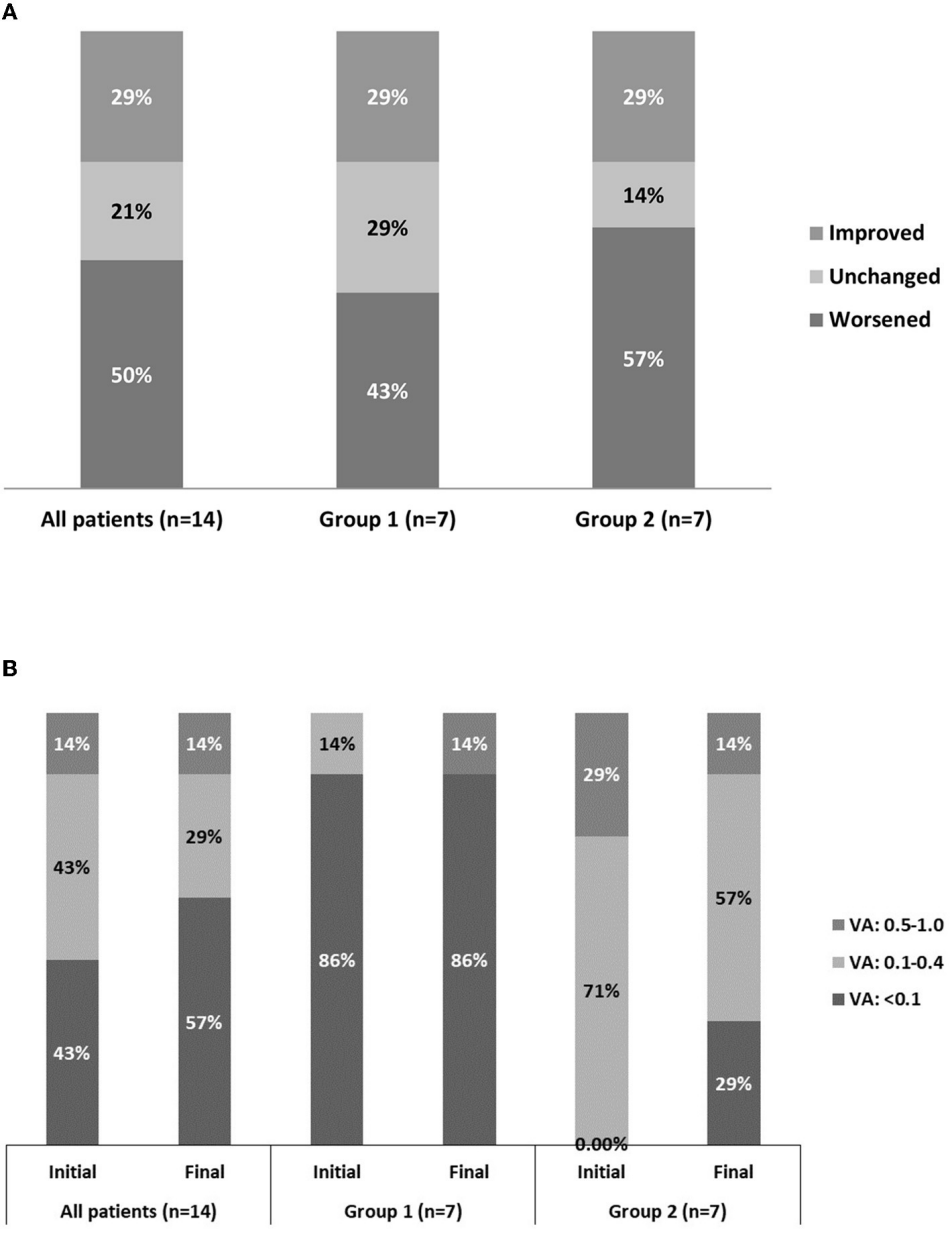
Fourteen eyes with documented initial vs. final visual acuity. Group 1 comprises the eyes in which orbital apex syndrome was attributed to neoplastic causes. Group 2 comprises the eyes in which orbital apex syndrome was attributed to non-neoplastic causes. **(A)** Categorization of visual improvement. **(B)** Categorization of visual acuity as good (0.5–1.0), fair (0.1–0.4), and poor (<0.1).

The initial visual acuity at diagnosis ranged from no light perception to 20/30, whereas the final visual acuity at the last follow-up visit ranged from no light perception to 20/20. Among the fourteen patients with documented final visual acuity, eight (57.1%) had a visual acuity worse than 20/200, in which five had no light perception. In patients with a final visual acuity worse than 20/200, malignancies accounted for 75% of the disease conditions. Among those with documented initial and final visual acuity, seven (50%) had worsened, three (21.4%) had stable, and four (28.6%) had improved visual acuity. Thorough statistical analysis by a paired *t*-test failed to demonstrate significant differences between initial visual acuity and post-treatment final visual acuity (*p* = 0.44) (Figures 2A,B). The reason for that Figure 2B shows different data is because only those with documented both initial and final visual acuity were included in the Figure 2B (which was fourteen patients out of all twenty patients). Among the fourteen patients, six patients had initial visual acuity <20/200, which was 43%, as shown in Figure 2B. The 0.0% in bar 2 in Figure 2B represents those with initial VA 0.5–1.0 in group 1 (neoplastic OAS). The 0.0% in bar 3 in Figure 2B represents those with initial VA <0.1 in group 2 (non-neoplastic OAS). Therefore, neoplastic causes seemed to correlate with poor visual acuity.

Our data show a relationship revealed by Fisher's exact test between the causes of the disease (neoplastic or non-neoplastic) and visual prognosis. Neoplastic causes tended to correlate with poor initial visual acuity (*p* = 0.035) and final visual acuity (*p* = 0.051).

The peripapillary retinal nerve fiber layer (RNFL) thickness was measured by optical coherence tomography in ten patients. Overall, the initial RNFL thickness was within 43 and 114 μm (mean ± SD: 80.7 ± 27.6 μm). The final RNFL thickness was documented in five patients with overall thickness within 30 and 113 μm (mean ± SD: 74.2 ± 34.4 μm). For those five patients with serial follow-ups of RNFL thickness, three patient demonstrated progressive thinning of RNFL thickness with an average of −7.7 μm decline (SD: 5.5 μm).

Automated perimetry was performed for visual field evaluation in case No. 2, 5, 6, 9, 10, 11, 17. Three patients demonstrated generalized depression, two showed paracentral scotoma. Case No. 6 presented as nasal defect respect the vertical midline, which was more likely attributed to his underlying history of subarachnoid hemorrhage due to head trauma.

### Mortality

During follow-up, mortality occurred in three (15%) patients, and the intervals between the onset of OAS and mortality were 6, 9, and 24 weeks, respectively. All mortalities documented in our study were attributable to underlying malignancies (Table 2).

**Table 2:** Details of clinical presentation, etiology, visual change, diagnostic tools and mortality in our case series.

**Items**	**Number (n)**	**Percentage (%)**
**Gender**		
Male	15	75
Female	5	25
**Laterality**		
Right eye	12	60
Left eye	8	40
**Original S/S**		
Ptosis	11	55
Proptosis	8	40
Diplopia	6	30
Blurred vision	16	80
Facial numbness	4	20
Headache	5	25
Others (red eye, eye pain)	2	10
**VA change**		
Better	4	20
Worse	7	35
No change	3	15
Others (no records)	6	30
**Diagnostic image tools**		
CT	8	40
MRI	5	25
CT/ MRI both	6	30
Loss to follow-up	1	5
**Mortality**		
Yes	3	15
No	16	80
Loss to follow-up	1	5

*VA, visual acuity; S/S, symptoms and signs; CT, computed tomography; MRI, magnetic resonance image*.

## Discussion

The literature on OAS related to ocular diseases is scarce and involves mostly small-scale studies, possibly due to the rarity of this disease entity (11–13) and heterogeneity of underlying etiologies that require experienced specialists for accurate diagnosis. The literature on OAS in Taiwan is limited to case reports only (14, 15). Therefore, our study collected more cases and aimed at providing a more general overview of the ophthalmic clinical features, etiologies, and visual prognosis of OAS.

The hallmarks of OAS are blurred vision and ophthalmoplegia. In our study, the most common complaints upon initial presentation were blurring of vision (80%), ptosis (55%), and proptosis (50%). The most common clinical signs included limited extraocular movement and positive relative afferent pupillary defects.

All our patients exhibited unilateral involvement of OAS, but bilateral involvement is still possible. Yusuke et al. reported a rare case of bilateral OAS caused by granulomatosis with polyangiitis (16). Although the limitation of extraocular movement was documented through a physical examination in 90% of the patients, only 30% reported subjective diplopia. This discrepancy might be due to poor visual acuity in the affected eyes, which failed to produce double vision; missing data due to the retrospective study design; or unreliable patient statements.

The underlying causes of OAS vary among inflammatory, neoplastic, traumatic, infectious, and vascular causes. Detailed clinical examination, appropriate imaging modalities, and sometimes tissue biopsy for histopathological examinations are crucial for establishing an accurate diagnosis. Aryasit et al. (16) reported 80 cases of OAS in which carotid-cavernous fistula was the leading cause, accounting for 37.5% of cases. Twenty-four (30%) patients had neoplasia, and the most common diagnosis was lymphoma (nine patients), followed by meningioma (seven patients). The results of our study are very different from those of previous studies in which malignant neoplasms accounted for over half of the cases. Furthermore, the neoplasms leading to OAS found in our study were mostly malignant, and direct local invasion from head and neck cancers accounted for the majority of cases. This disparity might be partly related to differences in the prevalence rates of certain malignancies in different countries. Taiwan has the highest incidence of oral cancer (17, 18) worldwide, and nasopharyngeal carcinoma (19) is more common in Asian populations.

Our study demonstrates that the overall visual prognosis of OAS is poor despite vigorous multidisciplinary management. More than half of our patients were legally blind, and only 28.6% experienced an improvement in visual acuity after treatment. A meta-analysis of traumatic OAS also revealed poor overall visual prognosis, with only 51.7% of patients experiencing improvement in vision despite various medical and surgical interventions (20). The even worse visual outcome in our study may be related to different etiologies of OAS, as the disease in more than half of our cases was attributed to neoplastic causes. In addition, our results showed that neoplastic causes of OAS correlated with poor initial and post-treatment visual prognosis (*p* = 0.035 and 0.051, respectively). There was no significant correlation between the initial and post-treatment visual acuity. While Aryasit et al. (16) reported no significant relationship between the causes of the disease and visual prognosis, there was no statistically significant relationship between the initial and post-treatment visual acuity.

In our study, mortality was documented in three patients, which occurred 6, 9, and 24 weeks after the diagnosis of OAS. All deaths were attributed to the underlying malignancies. Brain or skull metastases of cancers usually represent advanced disease stages with poor prognosis, and patients are often treated with palliative therapy. Prado-Ribeiro et al. (21) reported four cases of OAS caused by head and neck cancer, and the average survival after the diagnosis of OAS was 9.5 months. The incidence of OAS in cancer patients and its correlation with overall prognosis have not yet been established. However, our results clearly indicate that the occurrence of OAS can serve as an indicator of advanced disease status in cancer patients and may indicate foreseeable mortality.

Our study had several limitations. First, we frequently use Landolt C for visual acuity testing in Taiwan's hospitals because most patients fail to read English letters from Snellen chart. Second, due to the retrospective nature of our study, the pupil sizes or changes were not documented in the medical records. Patients with mydriasis due to compression of the oculomotor nerve were not found in our study. This important clinical manifestation should be carefully recorded in further large-scaled study. Third, this was a retrospective study; therefore, the issue of missing data was inevitably encountered. Since our study was based on data from a tertiary referral center, the patients tended to have greater disease severity and complexity, which may have contributed to the overall poor prognosis in our study. Therefore, the findings in our study population may not be generalizable to the entire population of Taiwan. Further studies involving multiple medical centers and larger sample sizes are necessary to overcome these limitations.

Nevertheless, our study is the first case series of OAS in Taiwan. We aimed to provide an overview of the typical clinical features, possible etiologies, potential management, visual prognosis, and overall prognosis of OAS. The factors related to poor visual outcomes were also addressed. We believe that our data can be beneficial to ophthalmologists, general practitioners, otolaryngologists, or even oncologists who are not familiar with this disease entity but need to be aware of the typical clinical manifestations, as some of the causes may lead to significant morbidity and mortality.

In conclusion, OAS may result from various etiologies, including traumatic, inflammatory, infectious, neoplastic, and vascular conditions. Our data showed that malignant neoplasms are the most common cause of OAS in Taiwan. Generally, most patients with OAS experience vision loss despite aggressive multidisciplinary management. In terms of visual acuity, patients with non-neoplastic causes may have more favorable outcomes. OAS caused by malignancies usually indicates late-stage underlying tumors and thus may indicate a poor systemic prognosis.

## Data Availability Statement

The original contributions presented in the study are included in the article/supplementary material, further inquiries can be directed to the corresponding authors.

## Ethics Statement

The studies involving human participants were reviewed and approved by Institutional Review Board of the National Cheng Kung University Hospital, Taiwan (IRB A-ER-109-077). Written informed consent for participation was not required for this study in accordance with the national legislation and the institutional requirements.

## Author Contributions

P-HL conceptualized and designed the study, drafted the initial manuscript, designed the data collection instruments, collected data, and carried out the initial analyses. S-CS had reviewed the literature and helped to edit the references. W-JL conceptualized and designed the study and reviewed and revised the manuscript. All authors approved the final manuscript as submitted and agree to be accountable for all aspects of the work.

## Funding

Half of publication fee will be funded by Chi Mei Medical Center after acceptance.

## Conflict of Interest

The authors declare that the research was conducted in the absence of any commercial or financial relationships that could be construed as a potential conflict of interest.

## Publisher's Note

All claims expressed in this article are solely those of the authors and do not necessarily represent those of their affiliated organizations, or those of the publisher, the editors and the reviewers. Any product that may be evaluated in this article, or claim that may be made by its manufacturer, is not guaranteed or endorsed by the publisher.
